# Targeting mantle cell lymphoma metabolism and survival through simultaneous blockade of mTOR and nuclear transporter exportin-1

**DOI:** 10.18632/oncotarget.16602

**Published:** 2017-03-27

**Authors:** Kazumasa Sekihara, Kaori Saitoh, Lina Han, Stefan Ciurea, Shinichi Yamamoto, Mika Kikkawa, Saiko Kazuno, Hikari Taka, Naoko Kaga, Hajime Arai, Takashi Miida, Michael Andreeff, Marina Konopleva, Yoko Tabe

**Affiliations:** ^1^ Department of Laboratory Medicine, Juntendo University Graduate School of Medicine, Tokyo, Japan; ^2^ Leading Center for the Development and Research of Cancer Medicine, Juntendo University Graduate School of Medicine, Tokyo, Japan; ^3^ Section of Molecular Hematology and Therapy, Department of Leukemia, The University of Texas MD Anderson Cancer Center, Houston, Texas, USA; ^4^ Laboratory of Proteomics and Biomolecular Science, Research Support Center, Juntendo University Graduate School of Medicine, Tokyo, Japan; ^5^ Department of Next Genertion Hematology Laboratory Medicine, Juntendo University Graduate School of Medicine, Tokyo, Japan

**Keywords:** mantle cell lymphoma, mTORC1/2, selective inhibitor of nuclear export, XPO1, metabolism

## Abstract

Mantle cell lymphoma (MCL) is an aggressive B-cell lymphoma with poor prognosis, characterized by aberrant expression of growth-regulating and oncogenic effectors and requiring novel anticancer strategies. The nuclear transporter exportin-1 (XPO1) is highly expressed in MCL and is associated with its pathogenesis. mTOR signaling, a central regulator of cell metabolism, is frequently activated in MCL and is also an important therapeutic target in this cancer. This study investigated the antitumor effects and molecular/metabolic changes induced by the combination of the small-molecule selective inhibitor XPO1 inhibitor KPT-185 and the dual mTORC1/2 kinase inhibitor AZD-2014 on MCL cells. AZD-2014 enhanced the KPT-185–induced inhibition of cell growth and repression of cell viability. The combination of KPT-185 and AZD-2014 downregulated c-Myc and heat shock factor 1 (HSF1) with its target heat shock protein 70 (HSP70). As a consequence, the combination caused repression of ribosomal biogenesis demonstrated by iTRAQ proteomic analyses. Metabolite assay by CETOF-MS showed that AZD-2014 enhanced the KPT-185–induced repression of MCL cellular energy metabolism through the TCA (Krebs) cycle, and further repressed KPT-185–caused upregulation of glycolysis.

Thus the simultaneous inhibition of XPO1 and mTOR signaling is a novel and promising strategy targeting prosurvival metabolism in MCL.

## INTRODUCTION

Mantle cell lymphoma (MCL) is an aggressive B-cell lymphoma characterized by the aberrant expression of several growth-regulating, oncogenic effectors and is frequently resistant to standard chemotherapy [1]. Although MCL is characterized by the t(11, 14)(q13;32) translocation that results in aberrant expression of cyclin D1 [2], cyclin D1 overexpression itself is not sufficient for development of MCL; additional genetic events are necessary [3]. Exportin 1 (XPO1) mediates nuclear export of numerous molecules, including oncogenic transcription factors, ribosomal subunits, and RNAs, and is critical for cancer survival and proliferation. Elevated expression of XPO1 has been reported in the hematologic and solid tumors [4], and we have reported that the overexpression of XPO1 is associated with poor clinical outcomes in MCL and AML [5, 6]. Small-molecule selective inhibitors of nuclear export (SINE), which specifically and irreversibly bind to the Cys528 residue in the cargo-binding groove of XPO1, block XPO1-dependent nuclear export [4]. We and others demonstrated that the SINE compound KPT-185 [7] blocks XPO1 function and exerts an anti-lymphoma effect on MCL cells by suppression of oncogenic mediators such as cyclin D1; repression of ribosomal biogenesis, and downregulation of translation/chaperone proteins [5, 8, 9]. However, we found that XPO1 inhibition by KPT-185 resulted in upregulation of glycolysis [9], which plays an important role in sustaining tumor growth [10]. The result allows us to develop combination strategies with other molecular-targeted agents. Cancer cell proliferation and oncogenesis are coupled to metabolic reprogramming [11], such as PI3K/AKT signaling to directly stimulate glycolytic metabolism [11]. Cancer cells are known to consume glucose at a higher rate than their normal counterparts and produce lactate rather than completely oxidizing the glucose-derived carbon, a phenomenon known as the Warburg effect [12].

PI3K/AKT/mTOR signaling plays an important role in regulating MCL cell growth and proliferation [13], especially mTOR signaling, which is known to control protein synthesis by inducing ribosome biogenesis and mRNA translation [14] and is a critical pathway in the biology of MCL [15]. mTOR exists in two functionally and structurally distinct complexes, mTOR complex 1 (mTORC1) and mTORC2. The essential core component of mTORC1 is RAPTOR (regulatory-associated protein of mTOR) and that of mTORC2 is RICTOR (rapamycin-insensitive companion of mTOR) [16]. Activated mTORC1 phosphorylates ribosomal S6 kinase (S6K), thereby activating it to subsequently phosphorylate ribosomal protein S6 and promote ribosome biogenesis, and further stimulates the metabolic pathways that ultimately drive cell growth [17]. The first-generation mTOR inhibitors, which selectively target mTORC1, have been disappointing in their efficacy and clinical activity [18] because of their inability to inhibit mTORC2, one of the potential key factors in rapamycin resistance. The selective targeting of mTORC1 by first-generation mTOR inhibitors results in cell survival and metabolic regulation via S6K1 and TSC1/2 activation, which are involved in a negative-feedback loop to regulate mTORC2 levels [19]. AZD-2014 [20] is a second-generation dual mTORC1/2 inhibitor currently being tested in clinical trials, including renal cancer [21] and advanced solid tumors [22]. AZD-2014 exhibits specific activity against mTORC1/2 and thereby efficiently blocks the AKT/mTOR signal transduction pathway [18] without negative-feedback induction of mTORC2 [23]. In addition, ATP-competitive inhibitors such as AZD-2014 more efficienly target mTORC1 and block phosphorylation of rapamycin-resistant mTORC1 outputs such as 4EBP1 phosphorylation at T37/46 (4EBP1-T37/46) and cap-dependent translation [24].

We reasoned that a SINE and an mTOR inhibitor together might have complementary anticancer propertiesand thus offer a rational therapeutic benefit in MCL. We therefore hypothesized that a combination of the XPO1 inhibitor KPT-185 andselective mTORC1/2 dual inhibitor AZD-2014 would demonstrate synergistic inhibition of MCL cell growth by repressing prosurvival metabolism in this cancer.

## RESULTS

### Synergistic anti-proliferative effects of KPT-185+AZD-2014 in MCL cells

**Figure 1:**
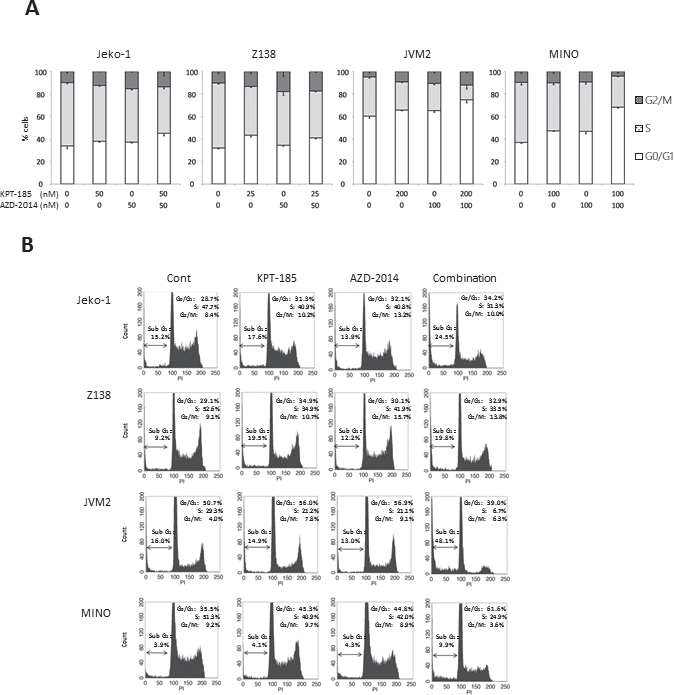
Induction of cell cycle arrest and apoptosis by the KPT-185 and AZD2014 combination in MCL cells Jeko-1, Z138, JVM2, and MINO cells were treated by indicated concentrations of KPT-185, AZD-2014, or KPT-185+AZD-2014 (combination) for 72 hours (Jeko-1, Z138, and JVM2) or 48 hours (MINO). The DNA contents were measured by flow cytometry. Graphs show the means ± SD of results of three independent experiments (**A**), and representative results show percentages of sub G_1_ cells (**B**). Cont; controls.

**Figure 2:**
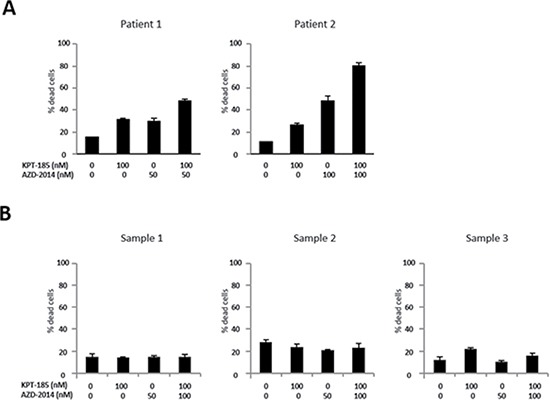
Cytotoxic effects of KPT-185 and AZD-2014 combination in primary MCL cells and not in normal hematopoietic stem cells Cells were treated by indicated concentrations of KPT-185, AZD-2014, or KPT-185+AZD-2014 (combination) for 24 hours. (**A**) Primary MCL cells from two patients were assessed by the trypan blue exclusion cell count method. (**B**) The annexin V positivity was measured by flow cytometry after electronic gating on CD34+ stem/progenitor hematopoietic cells from normal bone marrow mononuclear cells from three normal donors as described in Materials and Methods.

We first examined the effect of the XPO1 inhibitor KPT-185 and the mTORC1/2 inhibitor AZD-2014 on the proliferation of MCL cells. Four MCL cell lines were treated with KPT-185, AZD-2014, or a KPT-185+AZD-2014 combination for 48 hours. Both KPT-185 and AZD-2014 resulted in a dose-dependent reduction of cell viability as assessed by the CCK-8 assay (median inhibitory concentrations of KPT-185: 67 nM for Jeko-1, 41 nM for Z138, 684 nM for JVM-2, and 147 nM for MINO; of AZD-2014: 77 nM for Jeko-1, 108 nM for Z138, 179 nM for JVM-2, and 133 nM for MINO). The synergistic nature of the pharmacological interaction between KPT-185 and AZD-2014, which caused profound cell growth inhibition in all four cell lines, was shown by isobologram analysis (Table 1). Flow cytometric analysis of PI-stained cell nuclei showed that treatment with KPT-185+AZD-2014 decreased the number of cells in S phase, with concomitant G_0_/G_1_ phase accumulation, and increased the number of cells in sub-G_1_ phase compared to controls in all cell lines (Figure 1). We performed the additional KPT-185 combination experiments utilizing different mTORC1/2 inhibitors:, AZD-8055, a close analog of AZD-2014 [25] and a different chemotype MLN0128 [26], both of which demonstrated similar growth-inhibitory activity (Supplementary Figure 1). We further detected the profound cytotoxic effects of KPT-185 and AZD-2014 combination in MCL primary cells but not in CD34 positive cells from normal bone marrow samples (Figure 2).

**Table 1:** Combination indices for KPT-185+AZD-2014 in MCL cell lines

KPT-185+AZD2014	Jeko-1	Z138	JVM2	MINO
CI50	0.72	1.06	0.51	0.69
CI75	0.64	0.91	0.42	0.58
CI90	0.62	0.84	0.38	0.56
CI average	0.66	0.94	0.44	0.61

CI: combination index.

### Inhibition of ribosomal biogenesis through inactivation of HSF1 by KPT-185+AZD-2014 in MCL cells

To investigate alterations in biological processes induced by KPT-185 and AZD-2014, we employed a proteomic approach using iTRAQ to measure modulation of protein expression in response to treatment with KPT-185 and AZD-2014, either as monotherapy or in combination. A total of 1572, 2049, 2251, and 2375 unique proteins were identified in Jeko-1, Z138, JVM-2, and MINO cells, respectively, including 106, 142, 169, and 147 proteins whose expression level was significantly altered by KPT-185, AZD-2014, or KPT-185+AZD-2014.

To identify the alterations common to both KPT-185 and AZD-2014, proteins altered consistently in at least two of four tested cells were extracted. Expression of 32 proteins was consistently altered by KPT-185 (7 upregulated, 25 downregulated), 25 proteins by AZD-2014 (10 upregulated, 15 downregulated), and 54 proteins by KPT-185+AZD-2014(25 upregulated, 29 downregulated) (Supplementary Table 2).

Notably, 68% of the proteins downregulated by KPT-185 (i.e., 17 of 25) were ribosomal proteins, suggesting that KPT-185 strongly inhibited ribosomal biogenesis, a finding consistent with our previous report [9]. KPT-185 repressed the importin protein, regulator of nucleocytoplasmic protein transport including ribosomal proteins [27], along with XPO1. AZD-2014 downregulated elongation factors (EF2, EF1A1) and heat shock chaperone proteins (HSP90B, HSP7C). The KPT-185+AZD-2014 combination upregulated apoptosis-associated mitochondrial heat shock proteins and histone proteins.

We next examined the upstream regulators involved in the protein expression responses to KPT-185, AZD-2014, or KPT-185+AZD-2014 in the MCL cells by IPA (Table 2). The IPA platform highlighted repression of the multifaceted transcription factor heat shock factor 1 (HSF1) by KPT-185+AZD-2014 and by AZD-2014 alone; HSF1 is known to be a central transducer linking the translational activity of ribosomal biogenesis and transcriptional regulation of heat shock proteins [28]. Downregulation of the oncogenic transcription factor Myc, E2F1, and mTOR target p70S6K also was highlighted after KPT-185+AZD-2014 treatment. IPA ontology analysis of cDNA array data for Jeko-1 cells demonstrated downregulation of NOD2 (nucleotide-binding oligomerization domain containing 2)(Table 3), a member of the Nod1/Apaf-1 family that is known to trigger activation of MAP kinases and of NF-kappa-B signaling [29]. Indeed, MAPK14 and NFKB1 were moderately but significantly inactivated by KPT-185+AZD-2014 (z-score of IPA: MAPK14 -0.37, NFKB1 -0.3, *p* < 0.05). Whereas activation of tumor suppressor transcription factor TP53 has been observed after KPT-185+AZD-2014 treatment (Table 2, Table 3), mutant *TP53* bearing Jeko-1 and MINO cells showed no change of TP53 expression level by immunoblotting (data not shown). These results suggest that anti-tumor effects of KPT-185+AZD-2014 combination are not dependent on TP53 status, consistent with previous reports [8, 9].

**Table 2:** Upstream factors involved in protein expression responses to KPT-185, AZD-2014, or KPT-185+AZD-2014in MCL cells

Upstream Regulator	Activation z-score	*P*-value	Target molecules in dataset
KPT-185			
Upregulated			
RICTOR	3.606	5.88E-18	*PSME3, RPL10, RPL10A, RPL23, RPL26, RPL28, RPL4, RPL6, RPL7A, RPS10, RPS2, RPS23, RPS3*
TP53	1.432	8.79E-03	*HMGB2, HSPA8, KPNA2, PSME3, VIM, XPO1*
Downregulated			
MYC	−3.719	9.94E-12	*EEF2, LGALS1, RPL10, RPL19, RPL23, RPL26, RPL27, RPL35, RPL5, RPL6, RPL7A, RPS23, VIM, XPO1*
MYCN	−3.499	2.88E-26	*EEF2, LGALS1, RPL10, RPL19, RPL23, RPL26, RPL27, RPL28, RPL35, RPL4, RPL5, RPL6, RPS2, RPS23, RPS3, RPS4X, VIM*
HRAS	−1.982	6.54E-03	*HSPA8, RPS10, RPS3, VIM*
AZD-2014			
Downregulated			
HSF1	−1.387	1.17E-06	*CCT2, DNAJA1, HSP90AB1, HSPA8, HSPH1*
KPT-185+AZD-2014			
Upregulated			
TNF	2.795	2.54E-03	*FASN, HMGB1, HSP90AB1, HSPA8, HSPD1, PARP1, RRM1, RRM2, TPT1, VIM*
INSR	2.224	1.10E-07	*ACAA2, ATP5A1, CFL1, FASN, HSPD1, MDH2, PDCD4, RRM1, RRM2*
TP53	2.109	2.67E-07	*ACAA2, ADH5, FASN, HMGB1, HMGB2, HSP90AB1, HSPA8, HSPD1, KPNA2, MDH2, PSME3, RRM1, RRM2, VIM, XPO1*
PTEN	1.724	5.33E-05	*ACAA2, FASN, G3BP1, PARP1, PDCD4, PGK1, RRM1*
IFNG	1.387	2.38E-02	*EEF1A1, FASN, HMGB1, HSP90AB1, HSPA8, HSPD1, PSME3*
OSM	1.342	6.73E-03	*ADH5, FASN, KRT9, PDCD4, PGK1*
Downregulated			
p70S6k	−1.982	1.03E-09	*EEF1A1, EEF2, FASN, PDCD4, VIM*
E2F1	−1.673	1.21E-11	*CCT2, CSDE1, HMGB1, HMGB2, HNRNPD, HSPA8, HSPD1, HSPE1, KRT1, NCL, RRM1, RRM2, VIM*
EGF	−1.627	1.10E-04	*EEF1A1, FASN, NCL, PDCD4, RRM1, RRM2, VIM*
HSF1	−1.458	2.45E-10	*CCT2, DNAJA1, FASN, HMGB1, HSP90AB1, HSPA8, HSPD1, HSPE1, PGK1*
MYCN	−1.387	7.63E-15	*EEF1A1, EEF1G, EEF2, EIF4A1, HSP90AB1, HSPD1, NCL, RPL4, RPL6, RPS3A, RPS7, TPI1, VIM*

Data were analyzed by Ingenuity Pathway Analysis (IPA) based on the proteins whose expression was consistently altered after indicated treatment in Jeko-1, Z138, JVM2, and MINO cells (Supplementary Table 1).

**Table 3:** Upstream factors involved in transcriptional gene alterations by KPT-185, AZD-2014, or KPT-185+AZD-2014 in Jeko-1 cells

Upstream Regulator	Activation z-score
KPT-185+AZD-2014	
Upregulated	
TP53	1.534 ± 0.626
ESR1	1.959 ± 0.118
Downregulated	
NOD2	**−**1.510 ± 0.446

Data were analyzed by Ingenuity Pathway Analysis (IPA) based on the transcriptional genes that were consistently altered after indicated treatment inJeko-1 cells.

Values indicate the means ± SEMs of activation z-scores in two independent experiments.

**Figure 3:**
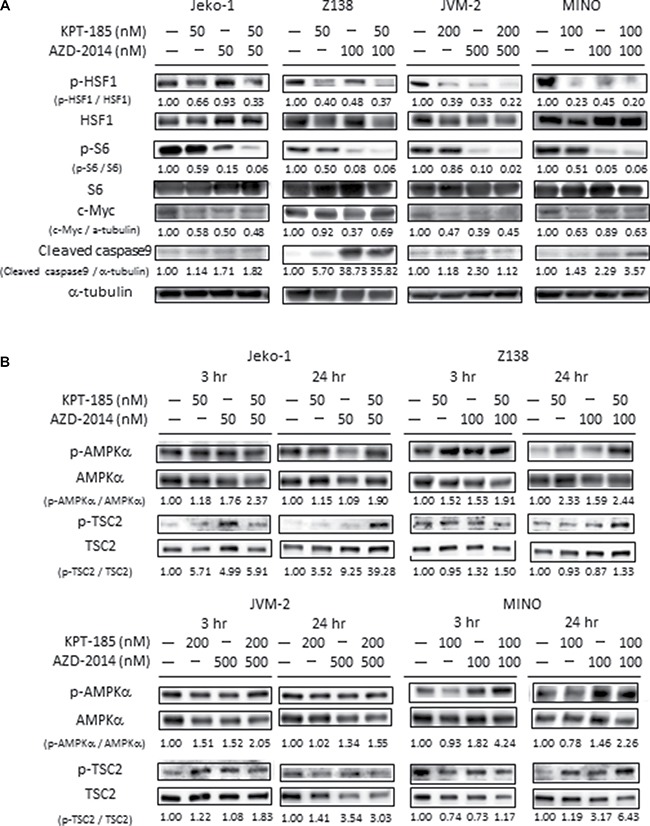
Molecular pathways affected by KPT-185 and AZD-2014 in MCL cells After treatment for 24 hours (**A**), and 3 or 24 hours (**B**) with KPT-185, AZD-2014, or KPT-185+AZD-2014 (combination) at indicated concentrations, the cells indicated were subjected to lysis and immunoblot analysis. The results are representative of three independent experiments, and the intensity of each immunoblot signal compared to that of α-tubulin was quantified using ImageJ software; the quantity is shown directly under each blot.

Immunoblot analysis demonstrated that AZD-2014 alone or combined with KPT-185 effectively suppressed p-S6 expression, and that the reduction of HSF1 phosphorylation and of c-Myc expression was caused by either KPT-185 and/or AZD-2014 in all MCL cells tested. (Figure 3A, Supplementary Figure 3A). Taken together, the XPO1 inhibition by KPT-185 and mTOR inhibition by AZD-2014 exhibited single-agent and/or combinatorial activities against the ribosomal biogenesis via inhibition of multiple factors including the transcription factor HSF1 and c-Myc.

### Inhibition of energy generation in MCL cells by KPT-185 and AZD-2014

KEGG pathway analysis based on the iTRAQ proteomic data highlighted downregulation of glycolysis/gluconeogenesis as the most significant pathway alteration by AZD-2014 alone and by KPT-185+AZD-2014 (*p* = 0.035). We examined the metabolome profiling of MCL cells after KPT-185, AZD-2014, or KPT-185+AZD-2014 treatment by CE-TOF-MS. A total of 93 and 56 metabolites were measured in Jeko-1 and Z138 cells, respectively (Supplementary Table 3). As expected from our previous findings [9], cells treated with KPT-185 showed higher levels of lactic acid than control cells. The KPT-185–induced upregulation of lactic acid was partially reversed by co-treatment with AZD-2014. We also observed decreases in levels of the tricarboxylic acid (TCA) or Krebs cycle metabolites, including citric acid, succinic acid, and malic acid, after treatment with single-agent KPT-185 or AZD-2014 Figure 4, decreases that were further abated by KPT-185+AZD-2014. In order to examine whether AZD-2014 induced suppression of glycolysis, adaptively increased in response to KPT-185, promotes cell cycle arrest and apoptosis, we next conducted the experiments using the combination of glycolysis inhibitor 2DG [30, 31] and KPT-185. The combined treatment with KPT-185 and 2DG caused cell growth inhibition in all four cell lines (Supplementary Figure 2A). Notably, the combination of 2DG with KPT-185 exhibited the profound effects on cell cycle arrest and apoptosis induction with decreased the number of cells in S phase, concomitant G0/G1 phase accumulation and accumulation of cells in sub-G1 phase, in the blastoid variant Z138 cells which is known to be highly proliferative and metabolically active [32–34], but only moderate to minimal effects in the classic typical MCL cells Jeko-1 [35], JVM-2 [34] and MINO [35]. (Supplementary Figure 2B).

**Figure 4:**
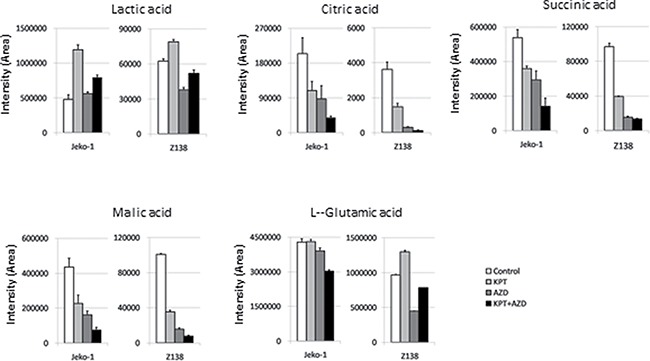
Quantification of metabolites affected by KPT-185, AZD-2014, or KPT-185+AZD-2014 The metabolites indicated were quantified inJeko-1 and Z138 cells treated with KPT-185, AZD-2014, or KPT-185+AZD-2014 (combination) for 18 hours (Jeko1:KPT-185 50 nM, AZD-2014 50 nM; Z138:KPT-185 25 nM, AZD-2014 50 nM) by CE-TOF-MS analysis. Graphs show the means ± SD of results in two independent experiments.

Because KPT-185 and AZD-2014 combination suppressed multiple pathways of energy production including glycolysis and TCA cycle, we investigated whether activity of the energy stress marker AMPK is modulated by KPT-185 and/or AZD-2014. We examined the phosphorylation levels of AMPKα and of tuberous sclerosis complex 2 (TSC2), a substrate of AMPK [19], at 3 and 24 hours after treatment. AMPKα phosphorylation was moderately increased by AZD-2014 in all tested cells at different time points, and was not clearly stimulated affected upon combination with KPT-185 (Figure 3B, Supplementary Figure 3C). On the other hand, KPT-185 and AZD-2014 combination increased TSC2 phosphorylation in Jeko-1 and MINO cells at 24 hour time-point. In JVM2 cells, AZD-2014 induced upregulation of phosphorylated TSC2 was not enhanced by KPT-185. Upregulation of phospho-TSC2 was observed in the blastoid variant Z138 by KPT-185 and/or AZD-2014 (Figure 3B, Supplementary Figure 3D). These results indicate that KPT-185 and AZD-2014 combination activates AMPK in a cell type-dependent manner.

## DISCUSSION

The results presented here demonstrate that simultaneous inhibition of XPO1 by KPT-185 and mTORC1/2 kinase by AZD-2014 effectively decreased growth of MCL cells and inactivated the TCA cycle and glycolysis. We previously reported that single-agent KPT-185 exhibited anti-proliferative and pro-apoptotic activities in MCL cells by repressing ribosomal biogenesis as well as inhibiting nuclear export of transcription factors and oncogenic mRNAs [9]. Intriguingly, however, the KPT-185–treated MCL cells exhibited upregulation of glycolysis and gluconeogenesis pathways [9], which may negatively affect the drug's antitumor activity. We therefore assessed the efficacy of an inhibitor of mTOR signaling, a central regulator of cell metabolism integrating nutrients, combined with KPT-185 targeting the altered metabolism.

It has been reported that cancer tissues express higher levels of lactic acid, TCA metabolites, and amino acids than normal tissues, which may reflect a cancer-specific energy metabolism mechanism to secure the continuous proliferation of cancer cells in the limited resources of their microenvironment [36]. Amino acids funnel into the TCA cycle [37] is favored as a source of energy in cancer cells. mTOR signaling is known to stimulate specific metabolic pathways, including glycolysis, the pentose phosphate pathway, and lipid biosynthesis [38].

Importantly, our data suggest that the KPT-185+AZD-2014 combination effectively inactivated energy metabolism of glycolysis and suppressed the TCA cycle in MCL cells. Although KPT-185 decreased the levels of TCA cycle metabolites, it did stimulate the breakdown of pyruvate to lactate. mTOR inhibition by AZD-2014 effectively reversed the KPT-185-induced activation of glycolysis, and promoted TCA cycle repression by KPT-185. We therefore propose that increased glycolysis might be an adaptive mechanism in response to KPT-185, which is, at least in part, suppressed by AZD-2014 to promote cell cycle arrest and death. The studies using glycolysis inhibitor 2DG combined with KPT-185 indeed demonstrated the cell growth inhibition in all examined cell lines with the most profound effects in the metabolically active blastoid variant MCL cells.

On the other hand, we observed that KPT-185 and AZD-2014 combination activated AMPK substrate TSC2 in the classic typical MCL cell lines but not in the blastoid variant MCL cells. The well-known clonal and genetic heterogeneity of MCL [2] could explain differential responses depending on MCL cell type seen in this study.

**Figure 5:**
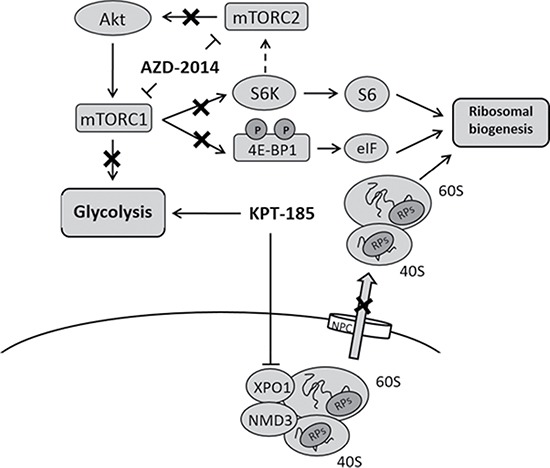
Combinational inhibition of XPO1 and mTOR impairs ribosomal biogenesis without glycolysis upregulation in MCL KPT-185 inhibits XPO1 mediating nucleocytoplasmic export of ribosomal subunits and increases glycolysis. Dual mTORC1/2 kinase inhibitor AZD-2014 represses S6K activation and 4EBP1 phosphorylation without negative-feedback induction of mTORC2, which results in decrease of ribosome biogenesis. mTOR inhibition by AZD-2014 suppresses glycolysis.

The antitumor effects of KPT-185 and AZD-2014 combination should be further confirmed in *in vivo* study. The multifaceted transcription factor HSF1, which was repressed by KPT-185+AZD-2014, coordinates net translational activity of ribosomal biogenesis and regulates a transcriptional network of genes driving heat-shock proteins, protein synthesis, and energy metabolism as a prime transducer [28]. Taken together, our findings may have important implications for combining mTOR kinase inhibitors with XPO1 antagonists for the treatment of MCL (Figure 5). The anticancer effects we observed suggest a novel, rationally designed combinatorial strategy targeting prosurvival metabolism in MCL.

## MATERIALS AND METHODS

### Cell cultures and reagents

Four representative MCL cell lines, Jeko-1 [39], Z138 [40], JVM-2 [41], and MINO [42], were used in this study. Z138 cell line has a profile of the blastoid variant MCL [32, 34]; Jeko-1, JVM-2 and MINO have the classic typical phenotypes [34, 35]. Z138 and JVM2 cells have wt-*TP53*, and the Jeko-1 and MINO cells harbor mutant *TP53* [35]. Cells were also treated with glycolysis inhibitor 2-Deoxy-D-glucose (2DG, Wako Pure Chemical Industries, Osaka, Japan) [30, 31], dual mTORC1/2 inhibitor AZD-8055 (LC laboratories, Woburn, MA) [25], MLN0128 (Selleck Chemicals, Houston, TX) [26]. Primary MCL samples and bone marrow mononuclear cells from healthy donors were obtained after informed consent in accordance with institutional guidelines set forth by Aichi Medical University and by MD Anderson Cancer Center per Declaration of Helsinki principles, respectively. MCL cell lines were cultured in RPMI 1640 medium containing 5% fetal bovine serum and 1% penicillin/streptomycin, primary MCL cells were cultured in MEMα medium containing 10% fetal bovine serum and 1% penicillin/streptomycin at 37°C in 5% CO_2_. Clinical characteristics of patients are summarized in Supplementary Table 1. Bone marrow mononuclear cells from healthy donors were seeded in StemSpan SFEM II (STEMCELL Technologies, Vancouver, BC, Canada), supplemented with BIT 9500 Serum Substitute (STEMCELL Technologies), cytokines including IL-3 (20 ng/ml), G-CSF (20 ng/ml), FLT3 ligand (50 ng/ml) and SCF (100 ng/ml) (all, Peprotech, Rocky Hill, NJ), StemReginin1 (Selleck Chemicals, Houston, TX) at 1 μM and 10-4 β-Mercaptoethanol [43, 44]. Cells were treated with SINE compound KPT-185 (provided by Karyopharm Therapeutics Inc., Natick, MA) [7], dual mTORC1/2 inhibitor AZD-2014 (Selleck Chemicals, Houston, TX) [20], or both at the indicated concentrations.

### Cell growth, apoptosis, and cell-cycle analysis

Proliferation of treated cells was determined by the CCK-8 colorimetric assay (Dojindo Molecular Technologies, Tokyo, Japan) according to the company's protocol. Apoptotic cell death and cell-cycle distribution in MCL cell lines were determined by flow cytometric analysis of propidium iodine (PI)-stained nuclei as described previously [45].

Cell death in primary MCL cells was assessed by the trypan blue exclusion cell count method [20]. Apoptosis in CD34+ stem/progenitor cells in normal bone marrow mononuclear cells was detected by flow cytometry using Annexin-V–APC (BD Biosciences, San Jose, CA), CD34-FITC (BD Biosciences) and DAPI. Data were analyzed by using Flowjo software (Tree Star, Ashland, OR).

### iTRAQ sample labeling, mass spectrometry analysis, and peptide identification

Proteins in treated cells were identified by isobaric tags for relative and absolute quantification (iTRAQ), a chemical labeling mass spectrometry (MS) method that was performed according to the manufacturer's protocol (AB SCIEX, Framingham, MA) [46, 47]. Briefly, the labeled peptides were analyzed by nano liquid chromatography in combination with tandem mass spectrometry (LC-MS/MS). Nano LC-MS was performed on nano LC system (AB SCIEX) using a ChromXP C18-CL column (Eksigent parts of AB SCIEX, Dublin, California, USA) and TripleTOF 5600 mass spectrometer for MS/MS (AB SCIEX) with Analyst TF 1.7 software. Protein identification and relative quantification were carried out by ProteinPilot Software Version 5.0 (AB SCIEX) [48]. The proteins identified were functionally defined by searching the UniProt database (Release 01/20/2016). Protein ratios were normalized against the overall median ratio for all the peptides in the sample for each separate ratio in every individual experiment. A confidence cutoff for protein identification of > 95% was applied. Proteins whose expression was statistically significantly changed by treatment were subjected to functional analysis by KEGG pathway enrichment analysis using DAVID Bioinformatics Resources [49] or the Ingenuity Pathway Analysis software (IPA, Ingenuity Systems, QIAGEN; www.qiagen.com/ingenuity) [9]. For IPA analysis, we used two scores: an ‘enrichment’ score (Fisher exact test *P*-value) that measures overlap of observed and predicted regulated gene sets and a Z-score that assesses the match of observed and predicted upregulation/downregulation patterns [50].

### Immunoblot analysis

Cells were solubilized in lysis buffer comprising phosphate-buffered saline solution containing 1× cell lysis buffer (Cell Signaling Technology, Danvers, MA), 1× protease inhibitor cocktail (Roche, Indianapolis, IN), and 1× phosphatase inhibitor cocktail (Roche) and incubated for 30 min on ice. The lysates were then subjected to centrifugation for 10 min at 13,000 rpm at 4°C. Total protein concentrations were determined by the Bio-Rad Protein Assay Kit (Bio-Rad Laboratories, Hercules, CA) according to the manufacturer's instructions. Total proteins (40 μg) were separated by sodium dodecyl sulfate–polyacrylamide gel electrophoresis (Bio-Rad Laboratories) and transferred to polyvinylidene-fluoride membranes (0.45 μm, Millipore, Bedford, MA), then probed with first and second antibodies according to the manufacturers’ protocols. The following antibodies were used: α-tubulin (Sigma-Aldrich, St Louis, MO), p-HSF1^Ser326^ (Abcam, Cambridge, MA), HSF1, p-S6 ribosomal protein (S6K) ^Ser235/Ser236^, S6 ribosomal protein, c-Myc, cleaved caspase-9, p-AMPKa^Thr172^, AMPKα, p-TSC2^Thr1462^, TSC2 and horseradish peroxidase–linked anti-mouse and anti-rabbit IgG (all, Cell Signaling Technology).

### Metabolite measurements

Metabolic extracts were prepared from cells (2–5 × 10^6^) by the HMT method (Human Metabolome Technologies, Inc., Tsuruoka, Japan) and analyzed on a capillary electrophoresis (CE)–quadruple-time of flight (Q TOF ) MS system (7100 capillary electrophoresis - 6530 Accurate Q TOF Mass; Agilent Technologies Inc. Santa Clara, CA). Briefly, the culture medium was removed from each dish, and the cells were washed twice in 5% mannitol solution (10 mL, then 2 mL). 1000μL of methanol containing internal standard (H3304–1002, Human Metabolome Technologies, Inc. Santa Clara, CA), 1000 μL of Chloroform and 400 μL of Milli-Q water were added to the cells and then centrifuged at 2,300 × g at 4°C for 5 minutes. Metabolite solutions were filtrated by Millipore 5-kDa cutoff filter at 9,100 × g at 4°C for 120 minutes and dried. The metabolites were resuspended in 50 μL of Milli-Q water containing internal standard (H3304–1004, Human Metabolome Technologies, Inc.) and applied to CE-MS. CE-MS experiments were carried out in positive and negative mode according to the methods developed by Soga et al [51, 52]. The obtained metabolite peaks were analyzed using Mass Profiler Professional (MPP) software (Agilent Technologies, Inc. Santa Clara, CA.)

### cDNA microarray

Gene expression in the cells was determined by microarray analysis using the Affymetrix Human Gene 2.0 ST Array according to Affymetrix protocols (Santa Clara, CA). Signal intensities were measured by using a GeneChip Scanner3000 7G (Affymetrix) and converted to numerical data by using the Affymetrix Expression Console Software 1.3.1 (Affymetrix). To identify candidate genes of potential significance in MCL, we applied a 1.5-fold change cutoff, since the combined responses of a group of genes acting in concert might affect the physiology of the cell, as previously described [53]. The digitized data were analyzed by GeneSpring GX 13.1.0 software (Agilent Technologies, Santa Clara, CA, USA).

### Statistical analyses

Groups were compared by a two-tailed Student *t*-test. A *P*-value ≤ 0.05 was considered statistically significant. Where indicated, the results are expressed as the mean ± standard deviation (SD) of triplicate samples. Synergism, additive effects, and antagonism were assessed by the Chou-Talalay method [54], utilizing Calcusyn software (Biosoft, Cambridge, UK). The effect on cellular proliferation was shown as a percentage reduction of cell viability when compared with dimethyl sulfoxide–treated controls. The average combination index (CI) value for the experimental combination was calculated from the 50%, 75%, and 90% effective doses (ED50, ED75, and ED90). By this method, CI values indicate the following: 0.3–0.7, strong synergism; 0.7–0.85, moderate synergism; 0.85–0.9, slight synergism; 0.9–1.1, nearly additive; 1.1–1.2, slight antagonism; 1.2–1.45, moderate antagonism; 1.45–3.3, antagonism; 3.3–10, strong antagonism [54].

We thank Kathryn Hale for manuscript review and Melodie England for help in the preparation of the manuscript.

## SUPPLEMENTARY MATERIALS FIGURES AND TABLES






