# Correction: Targeting mantle cell lymphoma metabolism and survival through simultaneous blockade of mTOR and nuclear transporter exportin-1

**DOI:** 10.18632/oncotarget.27309

**Published:** 2019-11-26

**Authors:** Kazumasa Sekihara, Kaori Saitoh, Lina Han, Stefan Ciurea, Shinichi Yamamoto, Mika Kikkawa, Saiko Kazuno, Hikari Taka, Naoko Kaga, Hajime Arai, Takashi Miida, Michael Andreeff, Marina Konopleva, Yoko Tabe

**Affiliations:** ^1^ Department of Laboratory Medicine, Juntendo University Graduate School of Medicine, Tokyo, Japan; ^2^ Leading Center for the Development and Research of Cancer Medicine, Juntendo University Graduate School of Medicine, Tokyo, Japan; ^3^ Section of Molecular Hematology and Therapy, Department of Leukemia, The University of Texas MD Anderson Cancer Center, Houston, Texas, USA; ^4^ Laboratory of Proteomics and Biomolecular Science, Research Support Center, Juntendo University Graduate School of Medicine, Tokyo, Japan; ^5^ Department of Next Genertion Hematology Laboratory Medicine, Juntendo University Graduate School of Medicine, Tokyo, Japan

**This article has been corrected:** Due to errors in image assembly, a western blot (TSC2) in Figure 3B was accidentally duplicated. The corrected Figure 3 is shown below. The authors declare that these corrections do not change the results or conclusions of this paper.

Original article: Oncotarget. 2017; 8:34552–34564. 34552-34564. https://doi.org/10.18632/oncotarget.16602

**Figure 3:**
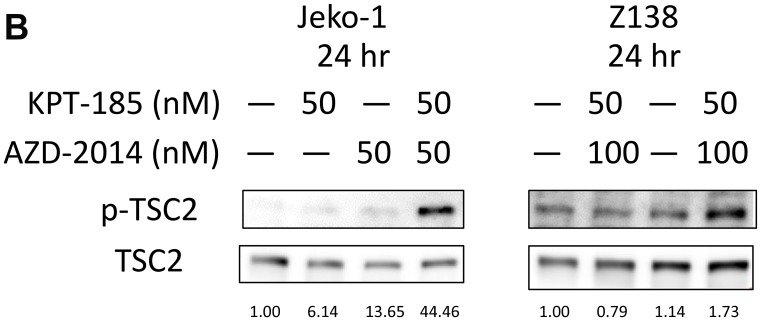
Molecular pathways affected by KPT-185 and AZD-2014 in MCL cells. After treatment for 24 hours (**A**), and 3 or 24 hours (**B**) with KPT-185, AZD-2014, or KPT-185+AZD-2014 (combination) at indicated concentrations, the cells indicated were subjected to lysis and immunoblot analysis. The results are representative of three independent experiments, and the intensity of each immunoblot signal compared to that of α-tubulin was quantified using ImageJ software; the quantity is shown directly under each blot.

