# Supplementary material for: Targeting mantle cell lymphoma metabolism and survival through simultaneous blockade of mTOR and nuclear transporter exportin-1
Source: Oncotarget. 2017 Mar 27;8(21):34552–64. doi: 10.18632/oncotarget.16602 (PMC5470990; doi:10.18632/oncotarget.16602)
Supplement: Supplementary file 1 [file oncotarget-08-34552-s001.pdf]

# Targeting mantle cell lymphoma metabolism and survival through simultaneous blockade of mTOR and nuclear transporter exportin-1

## Supplementary Materials

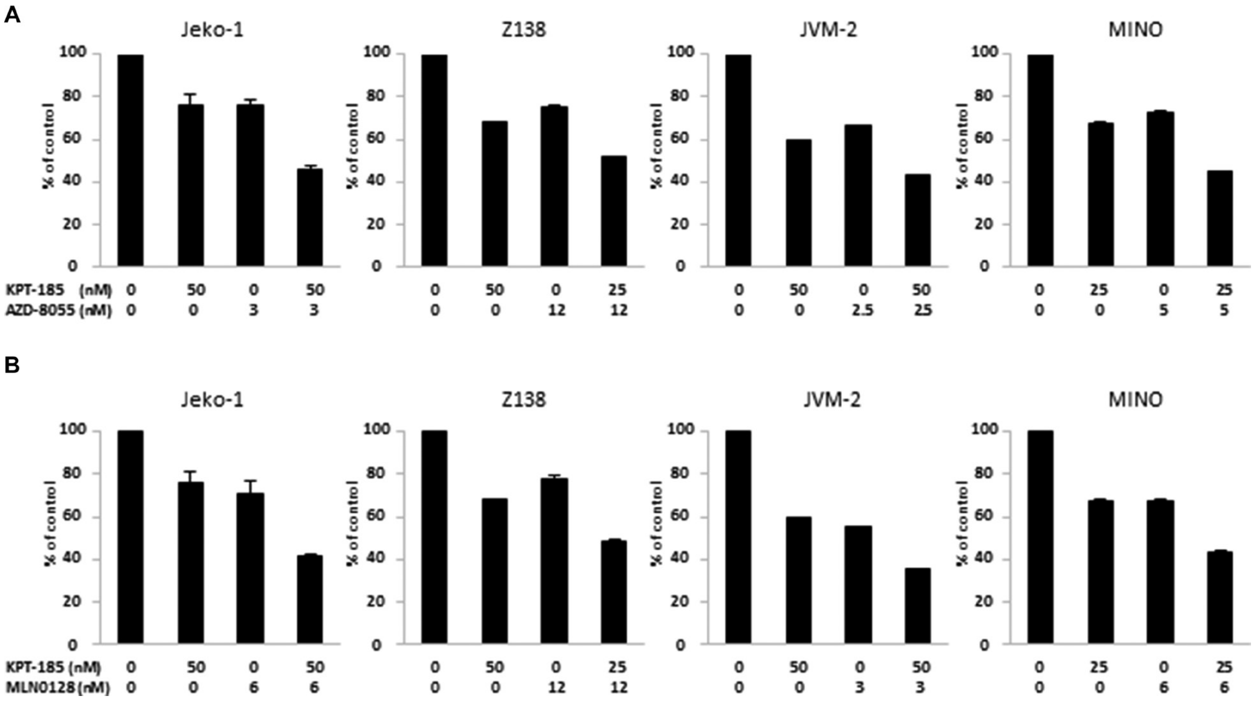


**Supplementary Figure 1: Cell growth inhibition by KPT-185 and AZD-8055 or MLN0128 combination in MCL cells.** Jeko-1, Z138, JVM2, and MINO cells were treated with indicated concentrations of KPT-185, AZD-8055, or KPT-185+AZD-8055 (combination) (**A**) and KPT-185, MLN0128, or KPT-185+MLN0128 (combination) (**B**) for 48 hours. Inhibition of cell growth is shown as percent of untreated control cells determined by the CCK-8 assay. Graphs show the means ± SD of the results of three independent experiments.

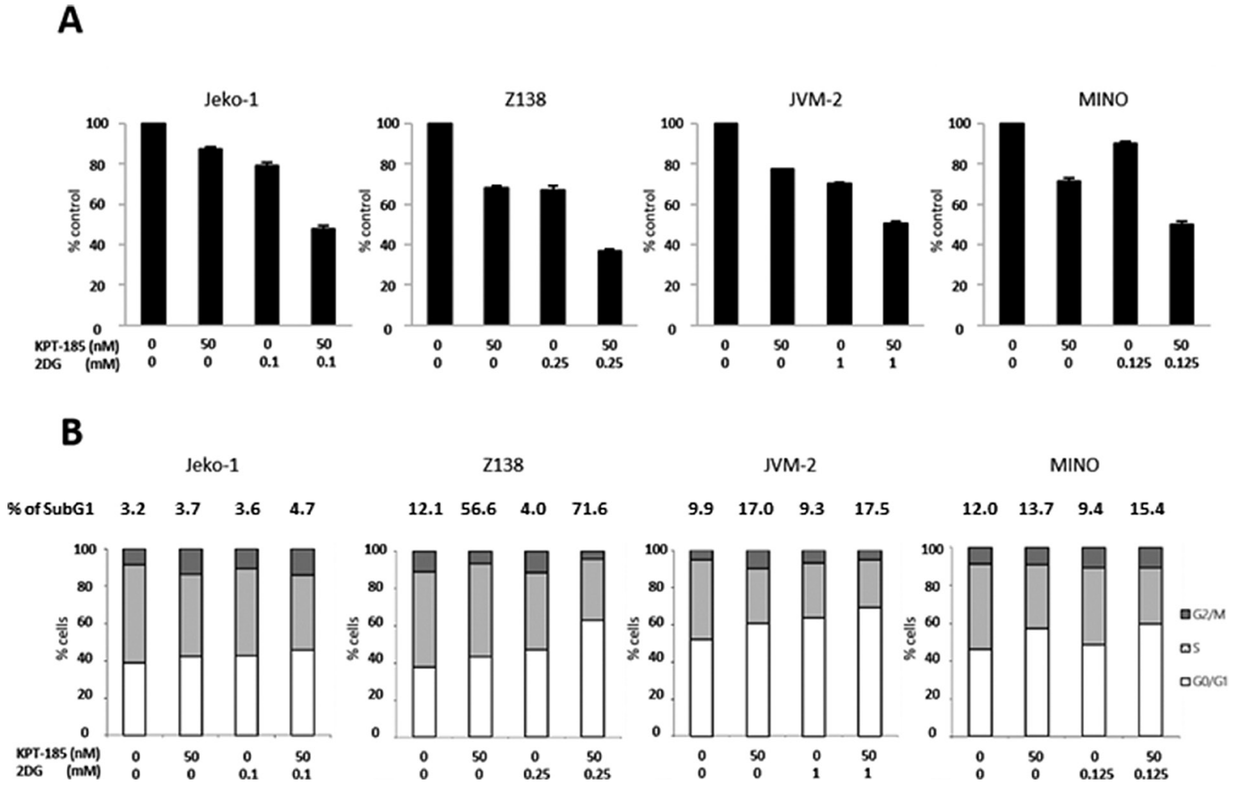


**Supplementary Figure 2: Induction of cell growth inhibition and cell cycle arrest by the KPT-185 and 2DG combination in MCL cells.** Jeko-1, Z138, JVM2, and MINO cells were treated with indicated concentrations of KPT-185, 2DG, or KPT-185+2DG (combination) for 72 hours. (**A**) Inhibition of cell growth is shown as percent of untreated control cells determined by the CCK-8 assay. (**B**) The DNA content was measured by flow cytometry. Graphs show the means ± SD of results of three independent experiments.

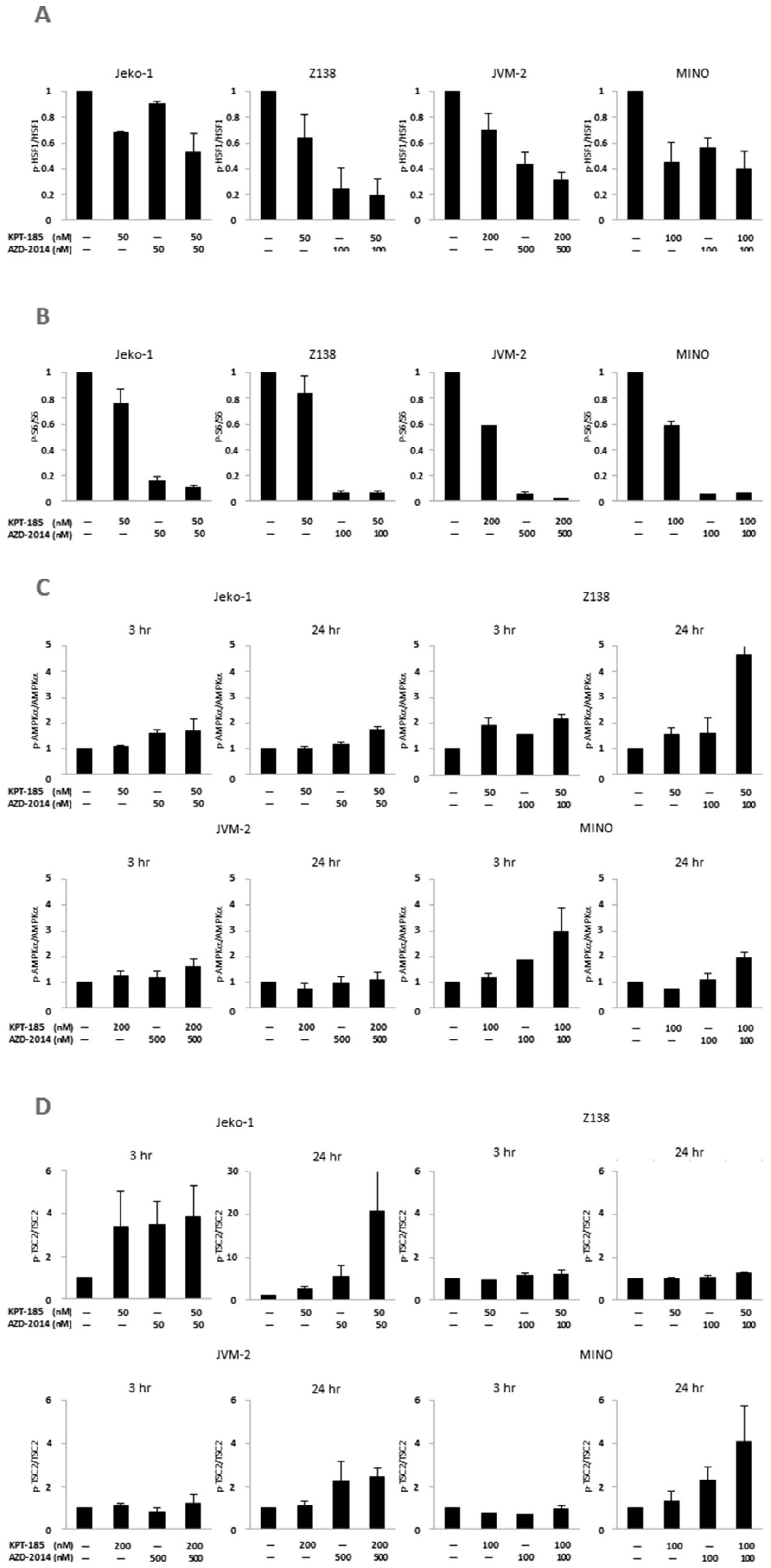


**Supplementary Figure 3: The expression of HSF1, S6, AMPK, and TSC2 in MCL cells treated with KPT-185 and/or AZD-2014.** The averaged p-HSF1 levels relative to HSF1; p-S6 levels relative to total S6; and p-AMPK levels relative to AMPK, and p-TSC2 levels relative to TSC2 after background subtraction. Graphs show the means ± SEM of the results of three independent experiments.

**Supplementary Table 1: Clinical characteristics of MCL patients**

| Patient number | source | age/sex | Cytogenetics (FISH) |
|---|---|---|---|
| 1 | BM | 74/M | IGH/BCL-1 (CCND1) 59.8% |
| 2 | PB | 63/F | IGH/BCL-1 (CCND1) 34.0% |

**Supplementary Table 2: Frequently altered proteins in MCL cells after treatment with KPT-185, AZD-2014, or KPT-185+AZD-2014.** See Supplementary_Table_2

**Supplementary Table 3: Frequently altered metabolites in MCL cells after treatment with KPT-185, AZD-2014, or KPT-185+AZD-2014.** See Supplementary_Table_3
