# Supplementary material for: Targeting mantle cell lymphoma metabolism and survival through simultaneous blockade of mTOR and nuclear transporter exportin-1
Source: Oncotarget. 2017 Mar 27;8(21):34552–64. doi: 10.18632/oncotarget.16602 (PMC5470990; doi:10.18632/oncotarget.16602)
Supplement: Supplementary file 2 [file oncotarget-08-34552-s002.docx]

**Supplemental Table 2.** Frequently altered proteins in MCL cells after treatment with KPT-185, AZD-2014, or KPT-185+AZD-2014

| Gene Symbol | Protein Name | Fold Change | | |
| --- | --- | --- | --- | --- |
| KPT-185 |  |  |  |  |
| Upregulated |  |  |  |  |
| *H4 (3)* | Histone H4 | 1.77 | ± | 0.36 |
| *H15 (2)* | Histone H1.5 | 1.71 | ± | 0.44 |
| *LEG1 (2)* | Galectin-1 | 1.46 | ± | 0.10 |
| *H2B1N (2)* | Histone H2B type 1-N | 1.31 | ± | 0.04 |
| *AHNK (2)* | Neuroblast differentiation-associated protein AHNAK | 1.26 | ± | 0.18 |
| *HMGB2* | High mobility group protein B2 | 1.18 | ± | 0.02 |
| *GDIR2 (2)* | Rho GDP-dissociation inhibitor 2 | 1.15 | ± | 0.01 |
| Downregulated |  |  |  |  |
| *EF2 (4)* | Elongation factor 2 | 0.88 | ± | 0.03 |
| *RL5 (2)* | 60S ribosomal protein L5 | 0.87 | ± | 0.01 |
| *RS4X (2)* | 40S ribosomal protein S4, X | 0.85 | ± | 0.06 |
| *RS3 (2)* | 40S ribosomal protein S3 | 0.84 | ± | 0.01 |
| *HSP7C (4)* | Heat shock cognate 71 kDa protein | 0.83 | ± | 0.04 |
| *RS23 (2)* | 40S ribosomal protein S23 | 0.82 | ± | 0.01 |
| *RL26 (2)* | 60S ribosomal protein L26 | 0.82 | ± | 0.04 |
| *RL19 (2)* | 60S ribosomal protein L19 | 0.82 | ± | 0.00 |
| *RS10 (2)* | 40S ribosomal protein S10 | 0.82 | ± | 0.06 |
| *RS2 (2)* | 40S ribosomal protein S2 | 0.82 | ± | 0.03 |
| *RL23 (2)* | 60S ribosomal protein L23 | 0.82 | ± | 0.02 |
| *RL4 (2)* | 60S ribosomal protein L4 | 0.81 | ± | 0.02 |
| *RL28 (2)* | 60S ribosomal protein L28 | 0.81 | ± | 0.00 |
| *RL10A (2)* | 60S ribosomal protein L10a | 0.80 | ± | 0.00 |
| *RL35 (2)* | 60S ribosomal protein L35 | 0.79 | ± | 0.02 |
| *RL27 (2)* | 60S ribosomal protein L27 | 0.79 | ± | 0.06 |
| *RL6 (2)* | 60S ribosomal protein L6 | 0.79 | ± | 0.04 |
| *PSME3 (2)* | Isoform 2 of Proteasome activator complex subunit 3 | 0.77 | ± | 0.10 |
| *RL7A (2)* | 60S ribosomal protein L7a | 0.77 | ± | 0.06 |
| *VIME (2)* | Vimentin | 0.76 | ± | 0.05 |
| *RL10 (2)* | 60S ribosomal protein L10 | 0.75 | ± | 0.03 |
| *XPO1 (2)* | Exportin-1 | 0.58 | ± | 0.14 |
| *IMA1(2)* | Importin subunit alpha-1 | 0.57 | ± | 0.08 |
| *K2C1 (2)* | Keratin, type II cytoskeletal 1 | 0.20 | ± | 0.02 |
| *K1C9 (2)* | Keratin, type I cytoskeletal 9 | 0.14 | ± | 0.03 |
|  |  |  |  |  |
| AZD-2014 |  |  |  |  |
| Upregulated |  |  |  |  |
| *PDCD4 (2)* | Programmed cell death protein 4 | 1.47 | ± | 0.15 |
| *H12 (2)* | Histone H1.2 | 1.46 | ± | 0.37 |
| *VDAC3 (3)* | Voltage-dependent anion-selective channel protein 3 | 1.34 | ± | 0.15 |
| *NPM (2)* | Nucleophosmin | 1.29 | ± | 0.16 |
| *HMGB2 (2)* | High mobility group protein B2 | 1.23 | ± | 0.06 |
| *ANXA5 (2)* | AnnexinA5 | 1.21 | ± | 0.00 |
| *RL12 (2)* | 60S ribosomal protein L12 | 1.18 | ± | 0.01 |
| *RL30 (2)* | 60S ribosomal protein L30 | 1.18 | ± | 0.01 |
| *PAIRB (2)* | Plasminogen activator inhibitor 1 RNA-binding protein | 1.14 | ± | 0.01 |
| *PARP1 (2)* | Poly [ADP-ribose] polymerase 1 | 1.10 | ± | 0.02 |
| Downregulated |  |  |  |  |
| *RS3A (2)* | 40S ribosomal protein S3a | 0.90 | ± | 0.52 |
| *TCPB (2)* | T-complex protein 1 subunit beta | 0.84 | ± | 0.02 |
| *HS90B (3)* | Heat shock protein HSP 90-beta | 0.83 | ± | 0.02 |
| *EF2 (4)* | Elongation factor 2 | 0.81 | ± | 0.05 |
| *PABP1 (2)* | Polyadenylate-binding protein 1 | 0.81 | ± | 0.01 |
| *HSP7C (4)* | Heat shock cognate 71 kDa protein | 0.80 | ± | 0.04 |
| *IF4G1 (2)* | Isoform 8 of Eukaryotic translation initiation factor 4 gamma 1 | 0.80 | ± | 0.04 |
| *HS105 (2)* | Heat shock protein 105 kDa | 0.79 | ± | 0.03 |
| *DNJA1 (2)* | DnaJ homolog subfamily A member 1 | 0.79 | ± | 0.03 |
| *YBOX1 (2)* | Nuclease-sensitive element-binding protein 1 | 0.79 | ± | 0.00 |
| *EF1A1 (2)* | Elongation factor 1-alpha 1 | 0.77 | ± | 0.02 |
| *TCTP (2)* | Translationally-controlled tumor protein | 0.74 | ± | 0.01 |
| *RIR1 (3)* | Ribonucleoside-diphosphatereductase large subunit | 0.73 | ± | 0.06 |
| *K2C1 (2)* | Keratin, type II cytoskeletal 1 | 0.34 | ± | 0.20 |
| *K1C9 (2)* | Keratin, type I cytoskeletal 9 | 0.25 | ± | 0.16 |
|  |  |  |  |  |
| KPT-185+AZD-2014 |  |  |  |  |
| Upregulated |  |  |  |  |
| *H2B1N (2)* | Histone H2B type 1-N | 1.91 | ± | 0.47 |
| *H4 (2)* | Histone H4 | 1.89 | ± | 0.36 |
| *PDCD4 (2)* | Programmed cell death protein 4 | 1.51 | ± | 0.04 |
| *NUCKS (2)* | Nuclear ubiquitous casein and cyclin-dependent kinase substrate 1 | 1.47 | ± | 0.26 |
| *H15 (2)* | Histone H1.5 | 1.44 | ± | 0.03 |
| *AHNK (2)* | Neuroblast differentiation-associated protein AHNAK | 1.33 | ± | 0.06 |
| *HMGB2 (4)* | High mobility group protein B2 | 1.32 | ± | 0.09 |
| *HNRPC (2)* | Heterogeneous nuclear ribonucleoproteinsC1/C2 | 1.27 | ± | 0.08 |
| *THIM (2)* | 3-ketoacyl-CoA thiolase, mitochondrial | 1.26 | ± | 0.02 |
| *HNRPD (2)* | Isoform 3 of Heterogeneous nuclear ribonucleoproteinD0 | 1.24 | ± | 0.05 |
| *MPCP (2)* | Isoform B of Phosphate carrier protein, mitochondrial | 1.21 | ± | 0.70 |
| *MDHM (4)* | Malate dehydrogenase, mitochondrial | 1.20 | ± | 0.08 |
| *ATPA (2)* | ATP synthase subunit alpha, mitochondrial | 1.20 | ± | 0.09 |
| *CH10 (2)* | 10 kDa heat shock protein, mitochondrial | 1.19 | ± | 0.02 |
| *HMGB1 (4)* | High mobility group protein B1 | 1.19 | ± | 0.05 |
| *ADHX (2)* | Alcohol dehydrogenase class-3 | 1.19 | ± | 0.02 |
| *HNRPU (2)* | Heterogeneous nuclear ribonucleoprotein U | 1.19 | ± | 0.03 |
| *AT5F1 (2)* | ATP synthase subunit b, mitochondrial | 1.18 | ± | 0.03 |
| *TOP1 (2)* | DNA topoisomerase 1 | 1.18 | ± | 0.01 |
| *NUCL (3)* | Nucleolin | 1.17 | ± | 0.04 |
| *TPIS (2)* | Triosephosphateisomerase | 1.16 | ± | 0.01 |
| *PGK1 (2)* | Phosphoglycerate kinase 1 | 1.15 | ± | 0.04 |
| *COF1 (2)* | Cofilin-1 | 1.13 | ± | 0.01 |
| *CH60 (2)* | 60 kDa heat shock protein, mitochondrial | 1.13 | ± | 0.00 |
| *PARP1 (2)* | Poly [ADP-ribose] polymerase 1 | 1.11 | ± | 0.02 |
| Downregulated |  |  |  |  |
| *EF1G (2)* | Elongation factor 1-gamma | 0.90 | ± | 0.00 |
| *RS3A (2)* | 40S ribosomal protein S3a | 0.88 | ± | 0.03 |
| *RL6 (2)* | 60S ribosomal protein L6 | 0.87 | ± | 0.05 |
| *IMDH2 (2)* | Inosine-5'-monophosphate dehydrogenase 2 | 0.87 | ± | 0.00 |
| *HS90B (3)* | Heat shock protein HSP 90-beta | 0.86 | ± | 0.06 |
| *EF1A1 (4)* | Elongation factor 1-alpha 1 | 0.85 | ± | 0.04 |
| *SYSC (2)* | Serine--tRNA ligase, cytoplasmic | 0.84 | ± | 0.06 |
| *RL4 (2)* | 60S ribosomal protein L4 | 0.84 | ± | 0.05 |
| *TCPB (2)* | T-complex protein 1 subunit beta | 0.82 | ± | 0.10 |
| *FAS (3)* | Fatty acid synthase | 0.82 | ± | 0.01 |
| *GUAA (2)* | GMP synthase [glutamine-hydrolyzing] | 0.81 | ± | 0.09 |
| *RS7 (2)* | 40S ribosomal protein S7 | 0.81 | ± | 0.03 |
| *EF2 (4)* | Elongation factor 2 | 0.80 | ± | 0.04 |
| *IF4G1 (4)* | Isoform 8 of Eukaryotic translation initiation factor 4 gamma 1 | 0.79 | ± | 0.06 |
| *NAA50 (2)* | N-alpha-acetyltransferase 50 | 0.79 | ± | 0.09 |
| *HSP7C (4)* | Heat shock cognate 71 kDa protein | 0.78 | ± | 0.03 |
| *IF4A1 (2)* | Eukaryotic initiation factor 4A-I | 0.78 | ± | 0.02 |
| *DNJA1 (3)* | DnaJ homolog subfamily A member 1 | 0.77 | ± | 0.03 |
| *TCTP (3)* | Translationally-controlled tumor protein | 0.77 | ± | 0.05 |
| *G3BP1 (2)* | RasGTPase-activating protein-binding protein 1 | 0.76 | ± | 0.06 |
| *RIR1 (2)* | Ribonucleoside-diphosphatereductase large subunit | 0.74 | ± | 0.10 |
| *IMA1 (2)* | Importin subunit alpha-1 | 0.74 | ± | 0.07 |
| *CSDE1 (2)* | Isoform 3 of Cold shock domain-containing protein E1 | 0.73 | ± | 0.04 |
| *PSME3 (2)* | Isoform 2 of Proteasome activator complex subunit 3 | 0.72 | ± | 0.08 |
| *VIME (2)* | Vimentin | 0.69 | ± | 0.07 |
| *RIR2 (2)* | Ribonucleoside-diphosphatereductase subunit M2 | 0.61 | ± | 0.00 |
| *XPO1 (2)* | Exportin-1 | 0.55 | ± | 0.21 |
| *K2C1 (2)* | Keratin, type II cytoskeletal 1 | 0.22 | ± | 0.05 |
| *K1C9 (2)* | Keratin, type I cytoskeletal 9 | 0.15 | ± | 0.04 |

The protein expression levels in Jeko-1, Z138, JVM2, and MINO cells were detected by iTRAQ. Expression of all proteins listed was significantly different (P<0.05) between controls and cells treated with KPT-185, AZD-2014, or KPT-185+AZD-2014. Values indicate fold-change relative to untreated cells. Confidence score (a percentage measure of the confidence of the protein identification) for all proteins in the table was 99%.

*(　): The number ofcell lines that that exhibited consistently altered expression levels after indicated treatment.
