# Supplementary material for: Targeting mantle cell lymphoma metabolism and survival through simultaneous blockade of mTOR and nuclear transporter exportin-1
Source: Oncotarget. 2017 Mar 27;8(21):34552–64. doi: 10.18632/oncotarget.16602 (PMC5470990; doi:10.18632/oncotarget.16602)
Supplement: Supplementary file 3 [file oncotarget-08-34552-s003.docx]

**Supplemental Table 3.** Frequently altered metabolites in MCL cells after treatment with KPT-185, AZD-2014, or KPT-185+AZD-2014

|  | Fold Change | | | | | | | | | | | |
| --- | --- | --- | --- | --- | --- | --- | --- | --- | --- | --- | --- | --- |
| Compound Name | Control | | | KPT-185 | | | AZD-2014 | | | KPT-185 + AZD-2014 | | |
| Jeko-1 |  |  |  |  |  |  |  |  |  |  |  |  |
| Cations |  |  |  |  |  |  |  |  |  |  |  |  |
| Spermidine | 1931 | ± | 606 | 2378 | ± | 581 | 3089 | ± | 525 | 1824 | ± | 502 |
| Putrescine | 1892 | ± | 700 | 3314 | ± | 675 | 2333 | ± | 364 | 1582 | ± | 458 |
| L(+)-Ornithine monohydrochloride | 30964 | ± | 2632 | 66254 | ± | 2927 | 50139 | ± | 1081 | 66284 | ± | 1900 |
| Choline base solution | 159419 | ± | 12877 | 152256 | ± | 9927 | 124541 | ± | 12022 | 97289 | ± | 10644 |
| L(+)-Lysine | 89550 | ± | 5243 | 115658 | ± | 3041 | 104316 | ± | 2051 | 102755 | ± | 4434 |
| S-(5'-Adenosyl)-L-methionine chloride | 75582 | ± | 3437 | 76433 | ± | 688 | 51058 | ± | 924 | 44157 | ± | 1261 |
| L(+)-Arginine | 402065 | ± | 15287 | 488557 | ± | 11243 | 521876 | ± | 8281 | 446785 | ± | 9554 |
| L-Histidine | 24442 | ± | 1807 | 39278 | ± | 952 | 30930 | ± | 868 | 41790 | ± | 2929 |
| Creatinine hydrochloride | 1302 | ± | 640 | 1743 | ± | 529 | 919 | ± | 275 | 1198 | ± | 163 |
| beta-Alanine | 15141 | ± | 353 | 17006 | ± | 331 | 11182 | ± | 1022 | 10575 | ± | 528 |
| Betainealdehyde+H2O | 38 | ± | 75 | 130 | ± | 150 | 0 | ± | 0 | 79 | ± | 91 |
| Adenine | 2713 | ± | 587 | 2436 | ± | 131 | 1754 | ± | 100 | 1231 | ± | 143 |
| 4-Aminobutyric acid (GABA) | 42281 | ± | 1010 | 45492 | ± | 2227 | 38375 | ± | 1040 | 37762 | ± | 417 |
| Tyramine | 33 | ± | 65 | 38 | ± | 77 | 0 | ± | 0 | 0 | ± | 0 |
| Glycine | 1042051 | ± | 27062 | 1058043 | ± | 23981 | 927604 | ± | 32009 | 778343 | ± | 24155 |
| Guanine | 31249 | ± | 1855 | 40566 | ± | 900 | 27552 | ± | 550 | 24521 | ± | 952 |
| Creatine monohydrate | 394336 | ± | 7130 | 421783 | ± | 11713 | 335437 | ± | 9351 | 300172 | ± | 9086 |
| L-Alanine | 533421 | ± | 23373 | 573022 | ± | 3261 | 559601 | ± | 13423 | 421174 | ± | 3218 |
| Sarcosine | 0 | ± | 0 | 0 | ± | 0 | 0 | ± | 0 | 0 | ± | 0 |
| Cytidine | 46103 | ± | 1590 | 49816 | ± | 2025 | 21986 | ± | 890 | 11955 | ± | 643 |
| Adenosine | 132 | ± | 186 | 168 | ± | 124 | 215 | ± | 189 | 155 | ± | 111 |
| L-Serine | 55428 | ± | 2107 | 108735 | ± | 666 | 76658 | ± | 1671 | 112840 | ± | 2652 |
| L-Valine | 65964 | ± | 616 | 84557 | ± | 2560 | 62899 | ± | 969 | 67986 | ± | 2307 |
| L-Homoserine | 0 | ± | 0 | 0 | ± | 0 | 0 | ± | 0 | 0 | ± | 0 |
| L(+)-Isoleucine | 89424 | ± | 3710 | 120370 | ± | 1679 | 94382 | ± | 1838 | 79927 | ± | 2034 |
| L-Leucine | 134043 | ± | 5598 | 169933 | ± | 7609 | 135362 | ± | 1384 | 125490 | ± | 1977 |
| L-Asparagine monohydrate | 535503 | ± | 7731 | 465180 | ± | 12228 | 583306 | ± | 8279 | 364140 | ± | 4934 |
| L(-)-Threonine | 173855 | ± | 2130 | 140461 | ± | 4743 | 187050 | ± | 2502 | 120820 | ± | 3623 |
| Anthranilic acid | 16646 | ± | 1707 | 11761 | ± | 1253 | 16448 | ± | 1877 | 8609 | ± | 772 |
| L-Methionine | 11025 | ± | 1390 | 14613 | ± | 2524 | 11540 | ± | 2014 | 9017 | ± | 1701 |
| L(+)-Glutamine | 154681 | ± | 2992 | 181017 | ± | 4513 | 239888 | ± | 4372 | 142430 | ± | 2024 |
| L(-)-Proline | 1115725 | ± | 19421 | 869824 | ± | 7094 | 1098166 | ± | 46853 | 664488 | ± | 12075 |
| L-Glutamic acid | 4280848 | ± | 152679 | 4307608 | ± | 98711 | 3910832 | ± | 121286 | 3033935 | ± | 56205 |
| L-Tryptophan | 13348 | ± | 656 | 16911 | ± | 660 | 12992 | ± | 1264 | 11243 | ± | 260 |
| Hypoxanthine | 146148 | ± | 2314 | 167231 | ± | 3770 | 133552 | ± | 2379 | 102773 | ± | 1792 |
| L-Citrulline | 3949 | ± | 215 | 5517 | ± | 246 | 7960 | ± | 886 | 13193 | ± | 519 |
| L(-)-Phenylalanine | 39380 | ± | 668 | 50267 | ± | 1642 | 40671 | ± | 946 | 39778 | ± | 836 |
| Betaine | 50325 | ± | 2970 | 52149 | ± | 1853 | 50280 | ± | 2217 | 38407 | ± | 1031 |
| L-Cysteine | 774 | ± | 852 | 817 | ± | 1069 | 623 | ± | 737 | 598 | ± | 612 |
| L(-)-Tyrosine | 31583 | ± | 1223 | 41065 | ± | 1346 | 33434 | ± | 1848 | 30823 | ± | 558 |
| L-Aspartic acid | 2020855 | ± | 46737 | 2454664 | ± | 14240 | 2240903 | ± | 49187 | 2283328 | ± | 31911 |
| L-Hydroxyproline | 421434 | ± | 7310 | 385271 | ± | 4130 | 432529 | ± | 12662 | 270679 | ± | 4525 |
| Glutathione (GSSG) | 42559 | ± | 3895 | 68951 | ± | 7238 | 41893 | ± | 5015 | 39432 | ± | 3629 |
| Glutathione (GSH) | 743310 | ± | 51353 | 395966 | ± | 54350 | 537284 | ± | 33708 | 262555 | ± | 38658 |
| Inosine | 3218 | ± | 381 | 3346 | ± | 435 | 5688 | ± | 369 | 4070 | ± | 254 |
| Thymidine | 9189 | ± | 1635 | 9572 | ± | 2215 | 8246 | ± | 2131 | 7670 | ± | 1402 |
| Uridine | 52372 | ± | 5950 | 55242 | ± | 6917 | 27494 | ± | 4013 | 20233 | ± | 2477 |
| Anion |  |  |  |  |  |  |  |  |  |  |  |  |
| NAD+ | 35563 | ± | 3392 | 39787 | ± | 3936 | 29371 | ± | 3556 | 27270 | ± | 3258 |
| Gluconic acid | 45615 | ± | 4700 | 39771 | ± | 1585 | 33462 | ± | 3068 | 26577 | ± | 2133 |
| GMP | 117991 | ± | 6612 | 96969 | ± | 8723 | 60444 | ± | 2006 | 45056 | ± | 2016 |
| AMP | 665907 | ± | 46435 | 620592 | ± | 35323 | 476284 | ± | 30231 | 390249 | ± | 15411 |
| NADP+ | 3587 | ± | 2413 | 7715 | ± | 1035 | 8347 | ± | 1333 | 10069 | ± | 1925 |
| 3-Hydroxybutyric acid | 740 | ± | 354 | 372 | ± | 287 | 118 | ± | 93 | 177 | ± | 58 |
| dTMP | 9813 | ± | 1110 | 8644 | ± | 732 | 5382 | ± | 766 | 2222 | ± | 177 |
| IMP | 105084 | ± | 3319 | 118808 | ± | 9108 | 92681 | ± | 5028 | 68152 | ± | 5257 |
| CMP | 65481 | ± | 3718 | 57569 | ± | 2319 | 37406 | ± | 2243 | 24833 | ± | 1924 |
| Sedoheptulose 7-phosphate | 2643 | ± | 366 | 616 | ± | 179 | 777 | ± | 408 | 588 | ± | 54 |
| 2-Hydroxybutyric acid | 30 | ± | 61 | 36 | ± | 73 | 31 | ± | 63 | 0 | ± | 0 |
| UMP | 293711 | ± | 17075 | 271656 | ± | 16821 | 145889 | ± | 8103 | 103858 | ± | 4384 |
| Glucose 6-phosphate | 1882 | ± | 150 | 1141 | ± | 682 | 402 | ± | 196 | 2343 | ± | 331 |
| Glucose 1-phosphate | 133 | ± | 114 | 72 | ± | 145 | 0 | ± | 0 | 0 | ± | 0 |
| Ribose-5-phosphate | 326 | ± | 105 | 247 | ± | 214 | 439 | ± | 148 | 707 | ± | 437 |
| Coenzyme A | 1683 | ± | 661 | 708 | ± | 414 | 2486 | ± | 507 | 498 | ± | 297 |
| GDP | 87353 | ± | 6761 | 79585 | ± | 11028 | 76203 | ± | 7268 | 69393 | ± | 6760 |
| Lactic acid | 479073 | ± | 66018 | 1188988 | ± | 69311 | 563803 | ± | 23029 | 793665 | ± | 34702 |
| ADP | 322731 | ± | 28649 | 355887 | ± | 53095 | 354124 | ± | 34997 | 355710 | ± | 27129 |
| Ribulose 5-phosphate | 1770 | ± | 264 | 1116 | ± | 43 | 1588 | ± | 292 | 1580 | ± | 487 |
| dTDP | 3510 | ± | 836 | 2740 | ± | 614 | 4252 | ± | 621 | 2178 | ± | 546 |
| GTP | 26350 | ± | 3460 | 26426 | ± | 6610 | 36334 | ± | 6020 | 39492 | ± | 8563 |
| CDP | 31042 | ± | 2364 | 32914 | ± | 3526 | 33963 | ± | 2440 | 30846 | ± | 3239 |
| Glyceraldehyde 3-phosphate | 1771 | ± | 265 | 1116 | ± | 42 | 1588 | ± | 292 | 1580 | ± | 487 |
| UDP | 139298 | ± | 10649 | 141279 | ± | 21024 | 130726 | ± | 12502 | 119977 | ± | 8176 |
| dATP | 62 | ± | 72 | 0 | ± | 0 | 435 | ± | 170 | 84 | ± | 58 |
| ATP | 157920 | ± | 11700 | 156550 | ± | 27200 | 241341 | ± | 10362 | 308775 | ± | 47913 |
| dTTP | 783 | ± | 194 | 566 | ± | 182 | 1758 | ± | 698 | 1076 | ± | 461 |
| Glycerol 3-phosphorate | 9793 | ± | 1413 | 4870 | ± | 641 | 4026 | ± | 534 | 3193 | ± | 802 |
| CTP | 12178 | ± | 1731 | 11440 | ± | 2360 | 16613 | ± | 2436 | 19304 | ± | 4082 |
| UTP | 62360 | ± | 6352 | 56800 | ± | 15405 | 71588 | ± | 9294 | 88910 | ± | 15519 |
| Pyruvic acid | 0 | ± | 0 | 6624 | ± | 1715 | 2759 | ± | 455 | 6751 | ± | 787 |
| Fructose 16-diphosphate | 2796 | ± | 441 | 1478 | ± | 798 | 1608 | ± | 152 | 1763 | ± | 430 |
| PRPP | 1202 | ± | 202 | 811 | ± | 374 | 1778 | ± | 235 | 1104 | ± | 333 |
| Succinic acid | 204025 | ± | 41463 | 108725 | ± | 23392 | 87368 | ± | 33945 | 39620 | ± | 3462 |
| Malic acid | 540083 | ± | 45537 | 358943 | ± | 14655 | 294478 | ± | 50949 | 141867 | ± | 46840 |
| Fumaric acid | 26625 | ± | 9336 | 17877 | ± | 5789 | 21892 | ± | 2600 | 12542 | ± | 1787 |
| Citric acid | 435506 | ± | 50845 | 226325 | ± | 47125 | 161152 | ± | 20981 | 73307 | ± | 16591 |
| Z138 |  |  |  |  |  |  |  |  |  |  |  |  |
| Cations |  |  |  |  |  |  |  |  |  |  |  |  |
| Spermidine | 819 | ± | 320 | 252 | ± | 85 | 1 | ± | 0 | 144 | ± | 94 |
| Putrescine | 4521 | ± | 364 | 2729 | ± | 421 | 5321 | ± | 525 | 1542 | ± | 113 |
| L(+)-Ornithine monohydrochloride | 15195 | ± | 1077 | 21005 | ± | 2404 | 15794 | ± | 223 | 26728 | ± | 797 |
| Choline base solution | 54914 | ± | 1212 | 46488 | ± | 2667 | 13284 | ± | 285 | 37504 | ± | 1429 |
| L(+)-Lysine | 73973 | ± | 1545 | 75560 | ± | 2129 | 28131 | ± | 932 | 45565 | ± | 796 |
| S-(5'-Adenosyl)-L-methionine chloride | 31749 | ± | 579 | 17678 | ± | 933 | 8126 | ± | 1008 | 8381 | ± | 366 |
| L(+)-Arginine | 196291 | ± | 1629 | 232092 | ± | 6263 | 98599 | ± | 5155 | 157452 | ± | 5358 |
| L-Histidine | 31273 | ± | 1039 | 25829 | ± | 1424 | 12465 | ± | 699 | 16130 | ± | 997 |
| Creatinine hydrochloride | 1853 | ± | 470 | 2556 | ± | 555 | 1161 | ± | 251 | 1663 | ± | 297 |
| beta-Alanine | 102389 | ± | 2726 | 65639 | ± | 3614 | 22363 | ± | 573 | 32125 | ± | 724 |
| Betainealdehyde+H2O | 0 | ± | 0 | 0 | ± | 0 | 0 | ± | 0 | 0 | ± | 0 |
| Adenine | 1035 | ± | 140 | 562 | ± | 355 | 280 | ± | 92 | 243 | ± | 89 |
| 4-Aminobutyric acid (GABA) | 24302 | ± | 1219 | 18869 | ± | 1288 | 7165 | ± | 229 | 10099 | ± | 447 |
| Tyramine | 0 | ± | 0 | 0 | ± | 0 | 0 | ± | 0 | 0 | ± | 0 |
| Glycine | 703849 | ± | 12191 | 677151 | ± | 6727 | 284555 | ± | 7583 | 437625 | ± | 7271 |
| Guanine | 1576 | ± | 227 | 2438 | ± | 294 | 458 | ± | 162 | 2504 | ± | 388 |
| Creatine monohydrate | 143996 | ± | 6167 | 138358 | ± | 9262 | 42017 | ± | 2912 | 67605 | ± | 5721 |
| L-Alanine | 232194 | ± | 5174 | 230509 | ± | 4025 | 103736 | ± | 2886 | 171418 | ± | 2726 |
| Sarcosine | 1 | ± | 0 | 1 | ± | 0 | 1 | ± | 0 | 1 | ± | 0 |
| Cytidine | 1729 | ± | 405 | 1538 | ± | 340 | 37 | ± | 73 | 812 | ± | 332 |
| Adenosine | 118 | ± | 79 | 1 | ± | 0 | 1 | ± | 0 | 72 | ± | 71 |
| L-Serine | 37012 | ± | 1168 | 60926 | ± | 1143 | 26266 | ± | 2300 | 34189 | ± | 1169 |
| L-Valine | 67647 | ± | 2228 | 57095 | ± | 2348 | 23301 | ± | 1334 | 31464 | ± | 3522 |
| L-Homoserine | 1 | ± | 0 | 1 | ± | 0 | 1 | ± | 0 | 1 | ± | 0 |
| L(+)-Isoleucine | 73728 | ± | 2080 | 62713 | ± | 1828 | 25418 | ± | 1448 | 35320 | ± | 878 |
| L-Leucine | 124107 | ± | 2133 | 97349 | ± | 4688 | 39344 | ± | 688 | 51079 | ± | 1991 |
| L-Asparagine monohydrate | 235815 | ± | 3997 | 197799 | ± | 2605 | 117062 | ± | 5018 | 140115 | ± | 4368 |
| L(-)-Threonine | 118050 | ± | 2204 | 104183 | ± | 2606 | 51656 | ± | 3737 | 66519 | ± | 674 |
| Anthranilic acid | 7770 | ± | 1092 | 4383 | ± | 1474 | 3496 | ± | 71 | 4043 | ± | 366 |
| L-Methionine | 9381 | ± | 911 | 13414 | ± | 1697 | 4440 | ± | 590 | 7343 | ± | 487 |
| L(+)-Glutamine | 10638 | ± | 1045 | 28576 | ± | 1002 | 11811 | ± | 1015 | 18984 | ± | 737 |
| L(-)-Proline | 867298 | ± | 5894 | 636636 | ± | 8965 | 338416 | ± | 11033 | 418684 | ± | 10194 |
| L-Glutamic acid | 967462 | ± | 9057 | 1297871 | ± | 20353 | 444004 | ± | 4187 | 781960 | ± | 3918 |
| L-Tryptophan | 20697 | ± | 1763 | 19278 | ± | 2160 | 11271 | ± | 2289 | 13437 | ± | 1921 |
| Hypoxanthine | 24172 | ± | 1296 | 23715 | ± | 504 | 10264 | ± | 632 | 26893 | ± | 975 |
| L-Citrulline | 11586 | ± | 910 | 13779 | ± | 697 | 8071 | ± | 658 | 11671 | ± | 553 |
| L(-)-Phenylalanine | 43340 | ± | 908 | 37860 | ± | 1662 | 13934 | ± | 504 | 18385 | ± | 51 |
| Betaine | 30411 | ± | 4395 | 17056 | ± | 2932 | 11795 | ± | 1215 | 30038 | ± | 1503 |
| L-Cysteine | 0 | ± | 0 | 0 | ± | 0 | 0 | ± | 0 | 0 | ± | 0 |
| L(-)-Tyrosine | 37474 | ± | 1336 | 30550 | ± | 435 | 12238 | ± | 614 | 16562 | ± | 966 |
| L-Aspartic acid | 1311433 | ± | 19495 | 1025574 | ± | 26810 | 611620 | ± | 20098 | 787600 | ± | 9824 |
| L-Hydroxyproline | 232958 | ± | 3925 | 151405 | ± | 2797 | 96805 | ± | 2430 | 102356 | ± | 2098 |
| Glutathione (GSSG) | 32029 | ± | 1118 | 25664 | ± | 2014 | 5641 | ± | 438 | 8342 | ± | 851 |
| Glutathione (GSH) | 239374 | ± | 7329 | 74384 | ± | 2247 | 45019 | ± | 2492 | 76565 | ± | 2596 |
| Inosine | 0 | ± | 0 | 0 | ± | 0 | 0 | ± | 0 | 0 | ± | 0 |
| Thymidine | 0 | ± | 0 | 0 | ± | 0 | 0 | ± | 0 | 0 | ± | 0 |
| Uridine | 0 | ± | 0 | 0 | ± | 0 | 0 | ± | 0 | 0 | ± | 0 |
| Anion |  |  |  |  |  |  |  |  |  |  |  |  |
| NAD+ | 0 | ± | 0 | 0 | ± | 0 | 0 | ± | 0 | 0 | ± | 0 |
| Gluconic acid | 7130 | ± | 104 | 3670 | ± | 385 | 1014 | ± | 122 | 1204 | ± | 81 |
| GMP | 15534 | ± | 130 | 10146 | ± | 297 | 2241 | ± | 260 | 4996 | ± | 320 |
| AMP | 146289 | ± | 2490 | 82270 | ± | 4138 | 35975 | ± | 312 | 49450 | ± | 2580 |
| NADP+ | 0 | ± | 0 | 0 | ± | 0 | 0 | ± | 0 | 0 | ± | 0 |
| 3-Hydroxybutyric acid | 0 | ± | 0 | 0 | ± | 0 | 0 | ± | 0 | 0 | ± | 0 |
| dTMP | 0 | ± | 0 | 0 | ± | 0 | 0 | ± | 0 | 0 | ± | 0 |
| IMP | 9482 | ± | 338 | 1862 | ± | 304 | 578 | ± | 58 | 1352 | ± | 99 |
| CMP | 5484 | ± | 170 | 6436 | ± | 399 | 915 | ± | 54 | 4521 | ± | 224 |
| Sedoheptulose 7-phosphate | 0 | ± | 0 | 0 | ± | 0 | 0 | ± | 0 | 0 | ± | 0 |
| 2-Hydroxybutyric acid | 0 | ± | 0 | 0 | ± | 0 | 0 | ± | 0 | 0 | ± | 0 |
| UMP | 25380 | ± | 340 | 15422 | ± | 645 | 4308 | ± | 84 | 9007 | ± | 252 |
| Glucose 6-phosphate | 0 | ± | 0 | 0 | ± | 0 | 0 | ± | 0 | 0 | ± | 0 |
| Glucose 1-phosphate | 0 | ± | 0 | 0 | ± | 0 | 0 | ± | 0 | 0 | ± | 0 |
| Ribose-5-phosphate | 0 | ± | 0 | 0 | ± | 0 | 0 | ± | 0 | 0 | ± | 0 |
| Coenzyme A | 0 | ± | 0 | 0 | ± | 0 | 0 | ± | 0 | 0 | ± | 0 |
| GDP | 9163 | ± | 513 | 9971 | ± | 411 | 1767 | ± | 204 | 4400 | ± | 134 |
| Lactic acid | 62387 | ± | 1938 | 78786 | ± | 2203 | 37871 | ± | 1874 | 51938 | ± | 2930 |
| ADP | 36979 | ± | 640 | 54130 | ± | 2071 | 19400 | ± | 491 | 31103 | ± | 1664 |
| Ribulose 5-phosphate | 0 | ± | 0 | 0 | ± | 0 | 0 | ± | 0 | 0 | ± | 0 |
| dTDP | 0 | ± | 0 | 0 | ± | 0 | 0 | ± | 0 | 0 | ± | 0 |
| GTP | 0 | ± | 0 | 0 | ± | 0 | 0 | ± | 0 | 0 | ± | 0 |
| CDP | 0 | ± | 0 | 0 | ± | 0 | 0 | ± | 0 | 0 | ± | 0 |
| Glyceraldehyde 3-phosphate | 0 | ± | 0 | 0 | ± | 0 | 0 | ± | 0 | 0 | ± | 0 |
| UDP | 0 | ± | 0 | 0 | ± | 0 | 0 | ± | 0 | 0 | ± | 0 |
| dATP | 0 | ± | 0 | 0 | ± | 0 | 0 | ± | 0 | 0 | ± | 0 |
| ATP | 8587 | ± | 425 | 31460 | ± | 780 | 9677 | ± | 794 | 17881 | ± | 753 |
| dTTP | 0 | ± | 0 | 0 | ± | 0 | 0 | ± | 0 | 0 | ± | 0 |
| Glycerol 3-phosphorate | 7434 | ± | 22 | 4818 | ± | 408 | 1279 | ± | 203 | 3193 | ± | 40 |
| CTP | 0 | ± | 0 | 0 | ± | 0 | 0 | ± | 0 | 0 | ± | 0 |
| UTP | 135 | ± | 0 | 4900 | ± | 334 | 168 | ± | 0 | 2521 | ± | 323 |
| Pyruvic acid | 0 | ± | 0 | 0 | ± | 0 | 0 | ± | 0 | 0 | ± | 0 |
| Fructose 16-diphosphate | 0 | ± | 0 | 0 | ± | 0 | 0 | ± | 0 | 0 | ± | 0 |
| PRPP | 0 | ± | 0 | 0 | ± | 0 | 0 | ± | 0 | 0 | ± | 0 |
| Succinic acid | 3623 | ± | 414 | 1486 | ± | 205 | 303 | ± | 38 | 143 | ± | 9 |
| Malic acid | 96573 | ± | 629 | 39594 | ± | 410 | 15689 | ± | 994 | 13632 | ± | 836 |
| Fumaric acid | 0 | ± | 0 | 0 | ± | 0 | 0 | ± | 0 | 0 | ± | 0 |
| Citric acid | 101026 | ± | 4114 | 35321 | ± | 1739 | 15578 | ± | 1530 | 7708 | ± | 938 |

The metabolitelevels were quantified by CE-TOF-MS analysis.

Values indicated the means ± SD of results in two independent experiments.
